# Gene expression associated with PTSD in World Trade Center responders: An RNA sequencing study

**DOI:** 10.1038/s41398-017-0050-1

**Published:** 2017-12-18

**Authors:** Pei-Fen Kuan, Monika A. Waszczuk, Roman Kotov, Sean Clouston, Xiaohua Yang, Prashant K. Singh, Sean T. Glenn, Eduardo Cortes Gomez, Jianmin Wang, Evelyn Bromet, Benjamin J. Luft

**Affiliations:** 1 Department of Applied Mathematics and Statistics, Stony Brook University, Stony Brook, NY, USA; 2 Department of Psychiatry, Stony Book University, Stony Brook, NY, USA; 3 Department of Family and Preventive Medicine, Stony Book University, Stony Brook, NY, USA; 4 Department of Cancer Genetics, Roswell Park Cancer Institute, Buffalo, NY USA; 5 Department of Biostatistics and Bioinformatics, Roswell Park Cancer Institute, Buffalo, NY USA; 6 Department of Medicine, Stony Brook University, Stony Brook, NY, USA

## Abstract

The gene expression approach has provided promising insights into the pathophysiology of posttraumatic stress disorder (PTSD). However, few studies used hypothesis-free transcriptome-wide approach to comprehensively understand gene expression underpinning PTSD. A transcriptome-wide expression study using RNA sequencing of whole blood was conducted in 324 World Trade Center responders (201 with never, 81 current, 42 past PTSD). Samples from current and never PTSD reponders were randomly split to form discovery (*N* = 195) and replication (*N* = 87) cohorts. Differentially expressed genes were used in pathway analysis and to create a polygenic expression score. There were 448 differentially expressed genes in the discovery cohort, of which 99 remained significant in the replication cohort, including *FKBP5,* which was found to be up-regulated in current PTSD regardless of the genotypes. Several enriched biological pathways were found, including glucocorticoid receptor signaling and immunity-related pathways, but these pathways did not survive FDR correction. The polygenic expression score computed by aggregating 30 differentially expressed genes using the elastic net algorithm achieved sensitivity/specificity of 0.917/0.508, respectively for identifying current PTSD in the replication cohort. Polygenic scores were similar in current and past PTSD, with both groups scoring higher than trauma-exposed controls without any history of PTSD. Together with the pathway analysis results, these findings point to HPA-axis and immune dysregulation as key biological processes underpinning PTSD. A novel polygenic expression aggregate that differentiates PTSD patients from trauma-exposed controls might be a useful screening tool for research and clinical practice, if replicated in other populations.

## Introduction

Posttraumatic stress disorder (PTSD) is a persistent and debilitating condition that affects approximately 7% of the US population^1^. Genetic vulnerability plays an important role in the etiology of PTSD. Twin and family studies indicate moderate heritability of PTSD^2–4^ and candidate gene studies have implicated a number of individual genes, such as *FKBP5* ^5,6^, *SLC6A4* ^7, 8^, *BDNF* ^9^, and *PACAP* ^10^. Although several studies have suggested that PTSD might be associated with a differential methylation pattern of some of these genes^11–14^, the largest epigenome-wide association study to date did not find any differentially methylated genes^15^. Altered gene expression has recently been viewed as a promising genetic process implicated in vulnerability to PTSD. Gene expression can help to identify critical downstream biological process through which the implicated genetic vulnerability is linked to the pathophysiology of the disorder and thus might inform efforts to identify potential PTSD biomarkers. The aim of the current study was to investigate whether PTSD is associated with an altered gene expression across the whole genome.

To date, there is a very limited knowledge about the genetic pathways leading to PTSD. As a result, the preferred method of explicating the pattern of gene expression underlying this disorder is the transcriptome-wide design, which allows for a thorough investigation of the expression patterns without relying on a priori knowledge of genetic risk factors^16^. Only a handful of transcriptome-wide gene expression studies of PTSD have been published to date^13,17–23^, most of which rely on small sample sizes (*N* ≤ 30). Of three notable exceptions, Mehta et al (2013) examined gene expression profiles of 169 trauma-exposed general population participants (61 with current PTSD) and found gene expression differences between PTSD cases and controls^13^. They also found downstream biological pathways enriched by these genes in PTSD, including pathways involved in cellular processes (e.g., cell migration and adhesion) and immunity (e.g., T cell activation). Logue et al (2015) examined 115 PTSD cases and 28 controls in a veteran sample, and identified 41 differently expressed genes, of which 7 remained significant in the replication sample, but only one (*ATP6AP1L*) survived the multiple-testing correction^17^. They also found that differentially expressed genes contributed to glucocorticoid signaling pathways. Breen et al (2016)^23^ examined 188 U.S Marines, constructed co-expression modules associated with PTSD, and found modules associated with hemostasis, interferon signaling and immune system. Taken together, the literature on the current transcriptome-wide expression studies in PTSD is small and inconclusive, with discrepancies possibly due to methodological differences in cohort characteristics, types of trauma and platforms. The existing studies are limited not only by their small sample sizes, but by their use of an older gene expression microarray approach. Only two studies^22,23^ utilized the more comprehensive RNA sequencing (RNA-Seq)^16^ platforms. Furthermore, none of the studies to date compared patients with current PTSD to remitted PTSD. Thus it is unclear whether altered gene expression reflects current symptoms or is an enduring vulnerability.

Both transcriptome-wide and, to a greater degree, candidate gene expression studies of PTSD have implicated differential expression of genes that play a role in the regulation of the glucocorticoid receptor in the glucocorticoid signaling pathway, most notably *BDNF* ^17,24–26^ and *FKBP5* ^19,21,27^. The glucocorticoid receptor plays a role in the regulation of the hypothalamic-pituitary-adrenal (HPA) axis, emotional memory formation and stress response processes^28,29^. Importantly, the glucocorticoid receptor also regulates the immune system^30^, in line with evidence that stressors can trigger an immune response^31,32^. Pre-existing vulnerabilities in glucocorticoid signaling have been identified in individuals with PTSD^30,33^. It is also well established that PTSD is associated with altered functioning of the immune system, including increased levels of circulating CRP and pro and anti-inflammatory cytokines^34–36^. Consistently, many of the genes regulating glucocorticoid receptor function that have been found to be differentially expressed in PTSD, such as *FKBP5*, have also been implicated in the immune response^6^. In sum, studies to date point to differentially expressed genes involved in the stress-induced glucocorticoid and immune system responses in PTSD.

The current study was designed to address the aforementioned limitations by identifying gene expression differences associated with PTSD across the entire genome using a hypothesis-free approach. Specifically, we conducted a transcriptome-wide expression study using the state-of-the-art RNA-Seq approach on RNA derived from whole blood. To this end, we recruited a large sample of participants (*n* = 324) who were exposed to a single traumatic event, the World Trade Center (WTC) disaster, thus reducing heterogeneity in the environmental exposure. Importantly, we used a non-overlapping replication sample within our cohort to validate significant results obtained in the discovery sample. To better understand genetic vulnerability to PTSD, we used gene expression findings to investigate biological pathways implicated in the disorder. A polygenic expression model to identify participants with PTSD was constructed using machine learning, tested in the replication sample, and used to compare current, past, and never PTSD.

## Methods

### Participants and clinical assessment

Participants were recruited through the Stony Brook WTC-Health Program^37^. The current study was approved by Stony Brook University IRB. Written informed consent was obtained. Inclusion criteria were sufficient English language skills to participate in a diagnostic interview, and being male. We included only males because females show notably different gene expression patterns from males^38^, and <10% of responders in the Stony Brook cohort were female. To insure adequate statistical power, we oversampled individuals with PTSD.

Master’s level psychologists were trained to administer PTSD module of the Structured Clinical Interview for DSM-IV (SCID^39^) with interval instructions (i.e., worst episode of symptoms since 9/11/2001). SCID items were modified to assess PTSD symptoms in relation to traumatic WTC exposures (Criterion A). Before conducting the assessment, the interviewers reviewed participants’ occupational and medical histories in order to facilitate rapport and enhance the accuracy of interpretation of responses. Inter-rater agreement for 55 independently rated audio-tapes was very good (kappa ≥ 0.82). Diagnoses were coded as (a) currently meets criteria for PTSD (current group), (b) met criteria since 9/11/2001 but did not meet currently (past group), and (c) did not meet criteria since 9/11/2001 (never group). The SCID was administered concurrently with the blood draw.

A total of 324 participants were profiled (201 never, 42 past, and 81 with current PTSD). We randomly split the current + never PTSD samples according to 7:3 ratio to form discovery and replication cohorts with sample sizes of 195 and 87, respectively. The 7:3 ratio is commonly used in data mining^40^. Splitting the data into discovery and replication is important in constructing and evaluating a prediction gene expression model (see subsection Polygenic expression score below). Additionally, participants completed Posttraumatic Stress Disorder Checklist-Specific Version (PCL-17)^41^, a 17-item self-report questionnaire assessing the severity of WTC-related DSM-IV PTSD symptoms in the past week. All of the participants were non-smoker, 87% were Caucasian, the mean age was 51.78 (SD = 8.12) (Table 1).

**Table 1:** Clinical characteristics of samples in discovery and replication cohorts

All	Current *N* = 81	Past *N* = 42	Never *N* = 201	P-value
Age				
Mean (SD)	52.94 (7.96)	51.57 (7.76)	51.36 (8.26)	0.331
Race N (%)				
Caucasian	69 (85.2)	33 (78.6)	181 (90.0)	0.100
Other	12 (14.8)	9 (21.4)	20 (10.0)	
**Discovery**	**Current** ***N = 57***		**Never** ***N = 138***	**P-value**
Age				
Mean (SD)	54.25 (7.82)		51.77 (8.46)	0.052
Race N (%)				
Caucasian	49 (86.0)		123 (89.1)	0.705
Other	8 (14.0)		15 (10.9)	
**Replication**	**Current** ***N = 24***		**Never** ***N = 63***	**P-value**
Age				
Mean (SD)	49.83 (7.57)		50.46 (7.78)	0.734
Race N (%)				
Caucasian	20 (83.3)		58 (92.1)	
Other	4 (16.7)		5 (7.9)	0.423

The *p*-values were computed from one way analysis of variance (for age in all samples), *t*-test (for age in discovery/replication cohort comparing current to never) and chi-squared test (for race)

### Whole genome transcriptome profiling via RNA-Seq

Gene expression of whole blood was profiled at the Roswell Park Cancer Institute Genomic Shared using RNA-Seq. For details on total RNA isolation and RNA-Seq library preparation see Supplementary Methods.

### RNA-Seq data preprocessing

Alignment was performed using the TopHat2 software^42^ which utilizes Bowtie2^43^ (http://bowtie-bio.sourceforge.net/bowtie2/index.shtml) on RefSeq (NCBI Reference Sequence Database) reference^44^ and annotation of the human genome (GrCh37-hg19 version). Other genomic related data was obtained using UCSC’s genome repository^45^. Quality control for the raw reads was performed with fastqc^46^, and adapter trimming was done with cutadapt^47^, Spliced alignment of the reads to the reference genome was done with the TopHat2 software allowing a maximum of one mismatch per read, and its quality control was done using RSeQC software^48^. The percentage of mapped reads ranged from 87.2 (88.8) to 97.3 (97.6) with a median of 92.6 (92.2) for the discovery (replication) samples. The number of counts mapping to each gene was computed^49^. We considered nascent and mature RNAs together (gene body counts) for the full view of the transcriptional landscape. Sensitivity analyses examined mature RNA alone (gene exon counts).

### Candidate SNPs genotyping

*FKBP5* polymorphisms have been shown to interact with PTSD symptom severity^27^ and childhood trauma in predicting PTSD^50^. Four SNPs (rs9296158, rs1360780, rs3800373, rs9470080) on *FKBP5* have been identified as risk alleles for PTSD^27,50^. SNP genotyping for these four SNPs was performed using Agena iPLEX assay (Agena Bioscience, San Diego, CA). For details on the protocol see Supplementary Materials. Risk alleles were A, T, G, and T in SNPs rs9296158, rs1360780, rs3800373 and rs9470080, respectively. Two sample *t*-tests were used to compare *FKBP5* normalized counts between risk and non-risk alleles for each SNP, stratified by PTSD status.

### Estimation of blood cell type proportions

Cell type proportions have been implicated in the analysis of whole blood samples. The proportions of CD8T, CD4T, natural killer, Bcell, monocytes and granulocytes were previously estimated in these samples^15^. Our prior study assayed DNA methylation on the Human Methylation 450 K BeadChip (Illumina Inc., San Diego, CA), and used R packages minfi and FlowSorted.Blood.450 to estimate blood cell type proportions based on the procedures described previously^52^. We normalized the sum of the proportions per sample to one, and include five out of six estimated cell types as adjustment factor in our differential expression analysis.

### Differential expression analysis

Differential expression analysis was performed using DESeq2^52^ software based on negative binomial generalized linear models, adjusting for age, race and the five cell type proportions (CD8T, CD4T, natural killer, Bcell, monocytes) in discovery and replication cohorts, respectively. Genes with low expression were filtered using the cpm (count-per-million) function in edgeR^53^. A total of 15192 genes were included in the analysis after filtering. Statistical significance was assessed via the Wald test. A false discovery rate (FDR)^54^ control was used to account for multiple testings. FDR < 0.05 was used to identify statistically significant genes from the discovery cohort. Nominal *p*-value < 0.05 were used to assess reproducibility of the differentially expressed genes in the replication cohort. The normalized read counts after adjustment for library sizes were used to generate boxplots.

### Candidate gene analysis

The association between current PTSD and gene expression was also examined for previously implicated genes. We used the same list of 27 genes compiled by Logue et al. (2015)^17^ and 8 additional genes they identified which were replicated (*p* < 0.05) in at least one of their two replication cohorts or the meta-analysis of the combined replication cohorts. Among these 35 candidate genes, 15 had low expression and were filtered from our data (Supplementary Table 2). The p-values from the discovery and replication cohort were combined using the weighted Stouffer’s method^55^ and multiplicity was adjusted via the Bonferroni method among these 20 genes.

### Pathway analysis

The Ingenuity Pathway Analysis (http://www.ingenuity.com/) was used to examine the functional pathways associated with the top ranking differentially expressed genes. Gene networks and canonical pathways representing key genes were identified using the curated ingenuity pathway analysis (IPA) database.

### Polygenic expression score

To evaluate the utility of transcriptome in identifying PTSD (current vs. never), the elastic net^56^ algorithm was applied to the discovery cohort using normalized counts.The elastic net was based on a regularized logistic regression model which automatically selected non-redundant informative genes in high-dimensional data to create a polygenic expression score, i.e., composite of genes that are most informative and predictive of PTSD status. The discovery and replication cohort served as training and test set, respectively. Separating the data into training and test set is important for evaluating the polygenic expression score in predicting PTSD status. The top ranking genes from the differential expression analysis in the discovery cohort were used as candidate feature set in the elastic net algorithm. The optimal tuning parameters were determined via a fivefold cross-validation. The area under receiver operating characteristics curve (AUC) and optimal cutoff based on Youden index *J* (defined as sensitivity + specificity–1) computed on the test set, i.e., replication cohort was used as metrics for performance evaluation. The sensitivity (Se), specificity (Sp), positive (PPV) and negative (NPV) predictive value at the optimal cutoff were also computed. Ability of the expression score to discriminate between cases and controls was tested in the replication sample, and resulting polygenic scores were also compared to the past PTSD group. Specifically, a linear model using the polygenic expression score as an outcome and the group membership as a covariate, adjusting for age, race and cell type proportions, was fitted. Spearman rank correlation was calculated to estimate the association between polygenic score and PCL in the replication sample.

An overview of the RNA-Seq data analysis pipeline was given in Supplementary Fig. 1. Additional statistical analyses were provided in Supplementary Materials.

### Data availability

The RNA-Seq data will be available at the Gene Expresion Omnibus (accession number GSE97356) upon publication.

## Results

### Participant characteristics

The PTSD groups did not differ significantly on age or race (Table 1). The genotypes for the four candidate SNPs (rs9296158, rs1360780, rs3800373, rs9470080) on *FKBP5* were not significantly associated with PTSD (Supplementary Table 5). Additional information on other clinical comorbidities were provided in Supplementary Table 7.

### Differentially expressed genes in discovery cohort and reproducibility in replication cohort

**Fig. 1:**
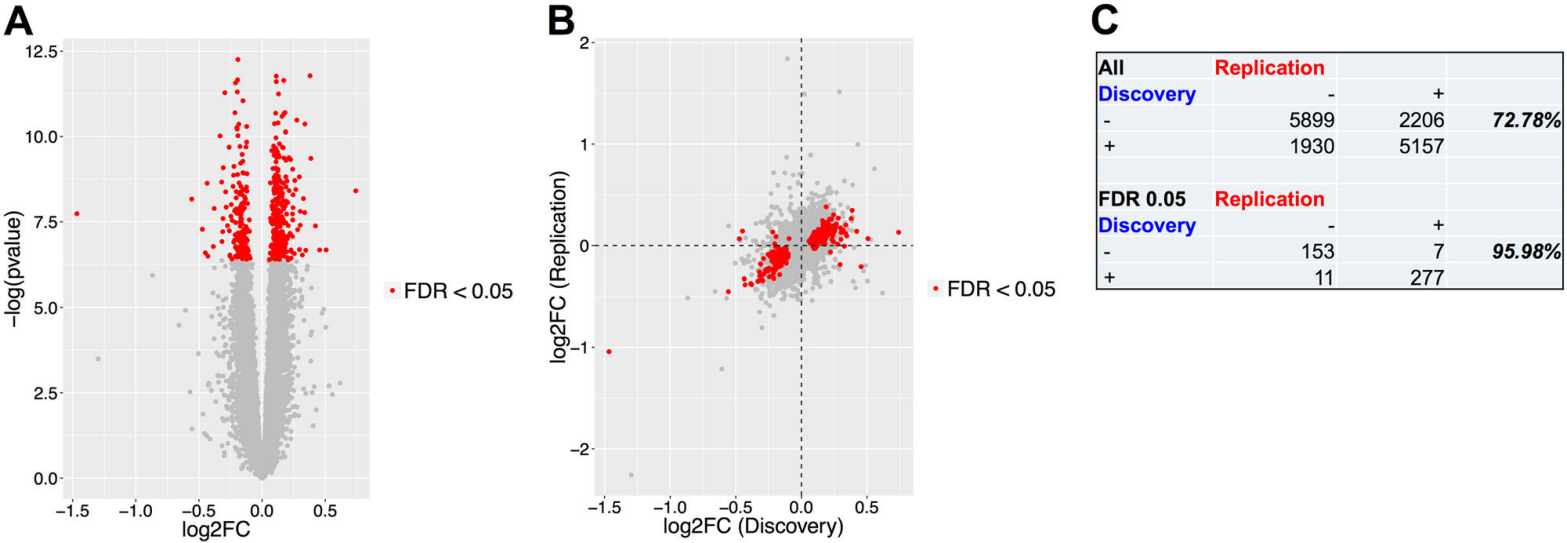
Differential expression analysis . **a**. Volcano plot displaying global differential expression patterns. **b**. Pair plot of estimated log2 fold change (FC) of discovery (*x*-axis) and replication cohort (*y*-axis). **c**. Percentage agreement in terms of sign of estimated log2 FC between discovery and replication cohort across all genes and genes at FDR < 0.05. Red dots correspond to the genes significant at FDR 0.05

**Fig. 2:**
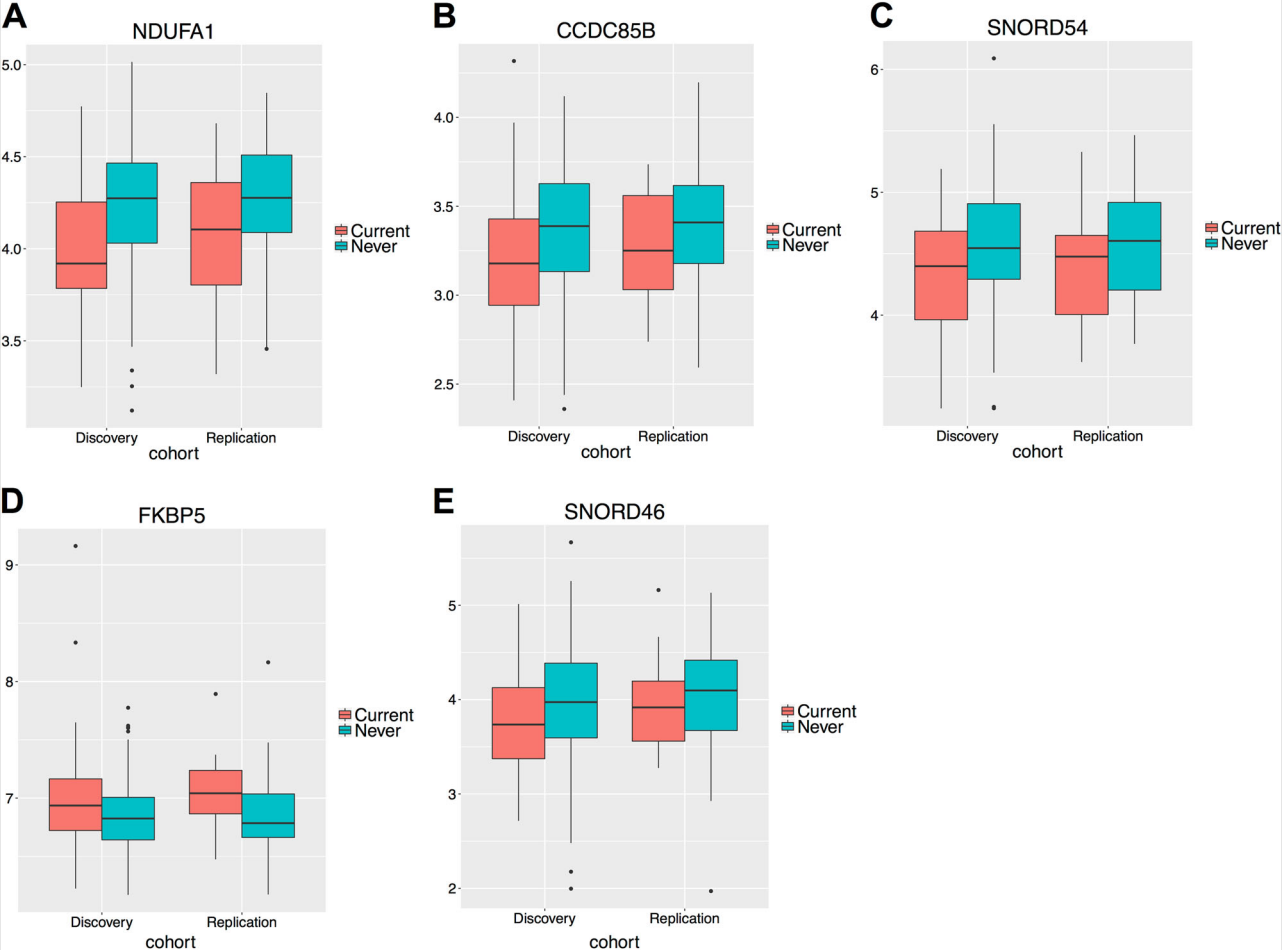
**a**–**e**. Boxplots of the log(normalized counts + 1) of NDUFA1, CCDC85B, SNORD54, FKBP5, and SNORD46, i.e., the five genes with FDR < 0.05, fold change > 1.2 in the discovery cohort, and nominal *p*-value < 0.05 in the replication cohort and consistent fold change estimate between discovery and replication cohort

The volcano plot (Fig. 1a) depicting global expression patterns indicates an approximately equal amount of up- and down-regulation in current compared to never PTSD. In total, 448 genes were differentially expressed at FDR < 0.05 (Supplementary Table 1) using counts which mapped to the gene body. The results from differential expression analysis were consistent between the discovery and replication cohort. Figure 1b displays the scatter plot of the estimated log2 fold change between the discovery (*x*-axis) and the replication (*y*-axis) cohort across all genes (Spearman rank correlation coefficient = 0.557). The correlation increased to *r* = 0.811 when we considered the 448 genes at FDR < 0.05. Among the 15192 genes, 11056 (72.8%) exhibited sign consistency in the estimated log2 fold change between the discovery and replication cohort (Fig. 1c). This number increased to 96.0% for the genes significant at FDR < 0.05. Among the 448 genes, 99 exhibited *p* < 0.05 in the replication cohort. Five of these 99 genes have absolute fold change > 1.2 and consistent fold change estimate, i.e., <5% difference in log2 fold change estimate between discovery and replication cohort. These genes were *NDUFA1*, *CCDC85B*, *SNORD54*, *FKBP5*, and *SNORD46* (boxplots Fig. 2a–e), of which all except *FKBP5* were down-regulated in current PTSD. *NDUFA1, CCDC85B*, and *FKBP5* remained significant when we considered exonic counts, whereas the majority of counts in *SNORD54* and *SNORD46* mapped to intronic regions. In addition, *FKBP5* gene expression were consistently up-regulated in current PTSD relative to never PTSD regardless of the genotypes. Although not statistically significant (*p* > 0.05), the mean *FKBP5* gene expression was lower in risk alleles compared to non-risk alleles for each of the four SNPs within current PTSD (Supplementary Fig. 4). From the candidate gene analysis, 5 of the 20 genes (*FKBP5, CASP2, SOD1, BBC3* and *C9orf84*) were significant at combined Bonferroni *p* < 0.05 (Supplementary Table 2). Additional results based on gene exon counts were provided in Supplementary Materials.

### Canonical pathway analysis

The top five canonical pathways among the 448 differentially expressed genes at FDR < 0.05 in the discovery cohort were the glucocorticoid receptor signaling pathway; pathway playing a role of macrophages, fibroblasts and endothelia cells in rheumatoid arthritis; actin cytoskeleton signaling pathway; NGF signaling pathway; and granzyme A signaling pathway (Table 2). These pathways have FDR 0.208 after adjusting for multiple comparisons. The complete ranked list of pathways is given in Supplementary Table 3. The glucocorticoid receptor signaling pathway was significant at FDR < 0.05 using results from gene exon counts.

**Table 2:** Top pathways identified by IPA among the 448 differentially regulated genes

Pathway	P-value	Overlapping genes
Glucocorticoid receptor Signaling	7.94E-04	PBRM1, PIK3CA, MED1, MAP3K1, JAK2, CEBPB, CD163, MED14, NCOA3, KAT2B, NFAT5, AKT1, PPP3CB, NCOR1, FKBP5
Role of macrophages, fibroblasts and endothelial cells in rheumatoid arthritis	1.38E-03	MAP2K6, PIK3CA, IL15, LTB, IRAK3, JAK2, PLCL2, CEBPB, ROCK2, ROCK1, TRADD, AKT1, NFAT5, PPP3CB, APC2
actin cytoskeleton signaling	2.14E-03	ROCK2, ROCK1, ABI2, PIK3CA, DIAPH2, PPP1R12A, ARPC5L, APC2, VAV3, PIP5K1B, TMSB10/TMSB4X, ARHGAP24
NGF signaling	2.63E-03	ROCK2, ROCK1, PIK3CA, AKT1, MAP3K1, RPS6KB2, RPS6KA3, BAX
Granzyme A signaling	4.68E-03	SET, HIST1H1E, H1FX

The Fisher’s *p*-value and the overlapping genes are provided.

### Polygenic expression score for PTSD status

**Fig. 3:**
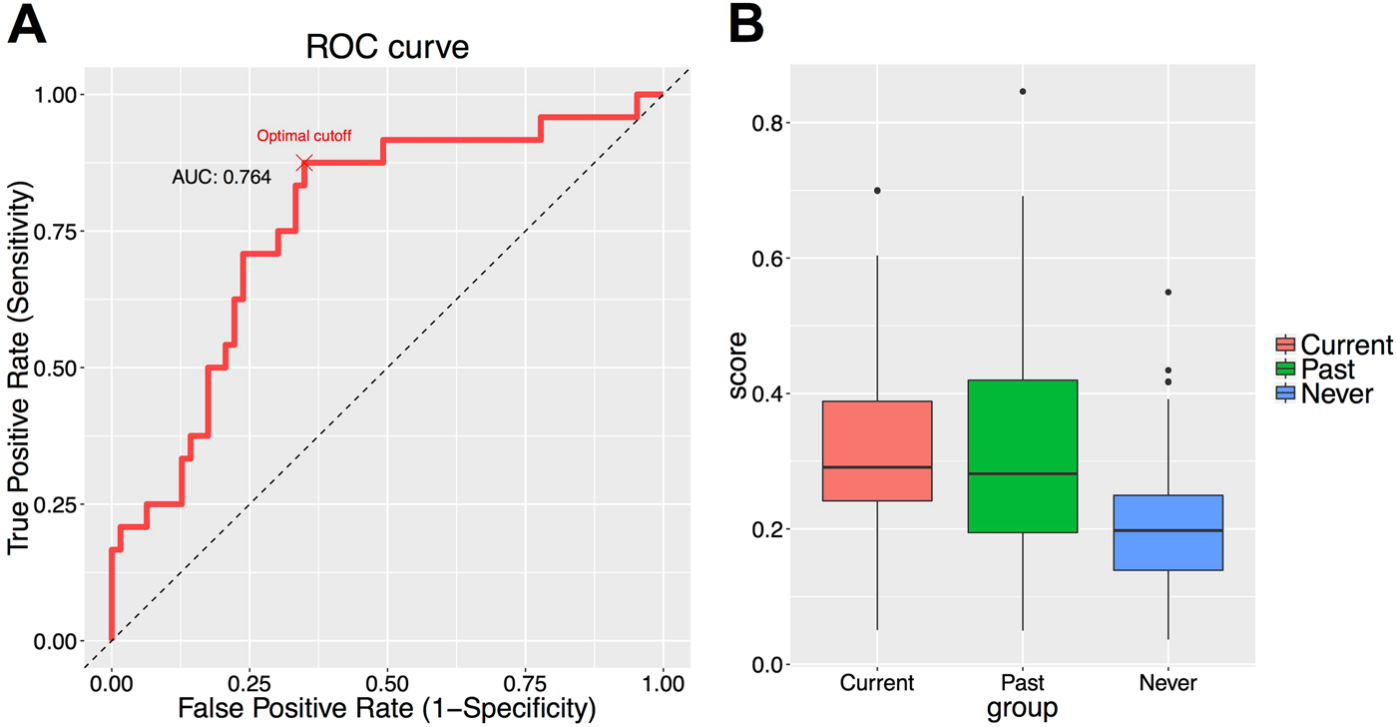
**a**. Receiver operating characteristic curve of the predicted PTSD probability on the replication cohort, trained using the 448 genes significant at FDR 0.05 on the discovery cohort via the elastic net prediction algorithm. **b**. Boxplot comparing the predicted risk score for current, past and never PTSD in the replication cohort

The polygenic expression score was trained on 448 genes at FDR < 0.05 from the discovery cohort. The final polygenic expression score from the elastic net algorithm retained 30 genes (Supplementary Table 4) and achieved AUC = 0.764 in the replication cohort (Fig. 3a). As a comparison, the gene with the largest AUC in the discovery cohort (training set) achieved AUC = 0.640 in the replication cohort, which indicated substantial improvement in PTSD prediction by aggregating multiple genes. The optimal Youden index *J* for the elastic net prediction model was 0.526 (sensitivity = 0.875, specificity = 0.651, positive predictive value = 0.488, negative predictive value = 0.932 at optimal cutoff 0.227). At cutoff 0.200, the prediction model achieved sensitivity = 0.917 and specificity = 0.508. On the other hand, the optimal Youden index *J* for the single gene predictor was only 0.276. Polygenic expression score was significantly correlated with PCL in the replication sample (*r* = 0.32, *p* < 0.01, Supplementary Fig. 3).

### Current and Past PTSD display comparable polygenic expression score

The polygenic expression score for current, past and never PTSD groups in the replication cohort is displayed in Fig. 3b. The past group exhibited the same level of expression score as the current group (*p* = 0.342), and the scores of each group were significantly different than the never group (*p* < 0.01 for both).

The comparison of the polygenic expression score vs. other clinical comorbidities were provided in Supplementary Materials. Additional results based on gene exon counts were provided in Supplementary Materials.

## Discussion

The current study compared transcriptome-wide gene expression of participants with current PTSD to trauma-exposed controls. We found 448 differentially expressed genes in the discovery cohort, out of which 99 remained significant in the replication cohort, and 5 had an estimated fold change magnitude > 1.2 and a consistent fold change estimates between discovery and replication cohorts, These 5 genes were *FKBP5, NDUFA1, CCDC85B, SNORD54*, and *SNORD46*. We also confirmed 5 out of 20 candiate genes: *FKBP5, CASP2, SOD1, BBC3*, and *C9orf84* that were previously found to be differentially expressed in PTSD^17^. Pathway analysis conducted using all 448 differentially expressed genes linked glucocorticoid receptor signaling, NGF signaling and several immunity-related pathways to PTSD. Furthermore, the polygenic expression score constructed using the aggregate of 30 differentially expressed genes provided a fair identification of participants with PTSD and indicated that the genetic signature was higher in both current and past cases as compared to controls. Taken together, the current study makes an important contribution to our understanding of the pattern of gene expression characterizing PTSD and biological pathways underpinning this disorder. These findings support earlier work implicating pathways linking the immune system with PTSD and, if independently confirmed, may inform development of novel diagnostic tools.

*FKBP5*, a gene that plays a role in the regulation of the glucocorticoid receptor and immunological responses to stress, was among our top most overexpressed genes in both our discovery and replication cohorts. Our findings are consistent with results of animal studies, which found increased expression of *FKBP5* in the brain after chronic exposure to stress and stress hormones^57–59^. In addition, *FKBP5* expression was also recently found to be up-regulated by exposure to stress hormones in humans^60^. We note, however, that three gene expression studies in humans found the reverse, reporting down-regulated *FKBP5* expression in PTSD^19,21,27^. This discrepancy might be due in part to sample differences in sex, age, and time since trauma, which were found to moderate HPA-axis function in PTSD^61,62^, and other as yet unidentified individual characteristics underlying the neurobiology of PTSD and *FKBP5* function specifically. Furthermore, the pattern of FKBP5 expression associated with PTSD depends on the functional polymorphisms within this gene, with down-regulation of *FKBP5* only found in carriers of the risk alleles^19,27^. We also found that the mean *FKBP5 expresion* was lower in the risk alleles among current PTSD in our sample. Collectively, the findings suggest that the role of *FKBP5* expression in the stress response is complex and determined by a number of other genetic and non-genetic factors. These complex mechanisms remain to be studied in large, heterogeneous samples.

Four other genes emerged as significantly differentially expressed in PTSD. First, we found down-regulation of *NDUFA1*, which has been implicated in neurodegenerative diseases^63,64^. *NDUFA1* is a gene responsible for respiratory electron transport in mitochondria and plays an important role in mitochondria DNA regulation^65^. Dysregulation resulting from missense mutation in *NDUFA1* contributes to Parkinson’s, Alzheimer’s and Huntington’s diseases. Second, we observed down-regulation of *CCDC85B*, a gene involved in p53 mediated regulation of β-catenin activity^66^. β-catenin has been implicated in neuronal synaptic plasticity and remodeling^67^. Altered β-catenin levels were observed in the hippocampus and amygdala of PTSD susceptible compared to PTSD resilient mice^68^. Furthermore, *CCDC85B* was identified as part of the gene expression regulation and inflammation networks associated with ADHD^69^. Finally, we identified down-regulation of two small nucleolar RNA (snoRNA) genes, *SNORD54* and *SNORD46. SNORD54* was found to be differentially expressed in autism spectrum disorder (AS)^70^, whereas expression of *SNORD46* was implicated in immune system function, specifically CD8 + T cells^71^, though not yet to psychiatric conditions per se. In addition, the majority of counts mapping to these two genes were in intronic regions, consistent with the findings that *SNORD*s function in pre-mRNA processing^72^. Overall, these findings point to differential expression of genes previously implicated in psychiatric and neurodevelopmental disorders that may also be critical to the etiology of PTSD, and if confirmed, may constitute novel genetic targets to be investigated for this disorder.

In aggregate, differentially expressed genes were found to contribute to several biological pathways that may play a role in PTSD, although none of the pathways remained significant after FDR correction. The glucocorticoid receptor signaling pathway emerged as a top pathway, which is in line with our *FKBP5* expression findings described above, which is a gene implicated in regulation of glucocorticoid receptor. The findings also link to the vast literature implicating the HPA-axis dysregulation in PTSD, which engages glucocorticoid receptor and is the major constituent of the neuroendocrine response to acute and chronic stress. Glucocorticoid signaling also regulates the immune system, thus it is not surprising that two immunity-related pathways emerged in the analyses: pathway involved in a role of macrophages, fibroblasts and endothelia cells in rheumatoid arthritis pathway, and the granzyme A signaling pathway. PTSD is consistently associated with heightened inflammation^34–36^, and it is also comorbid with autoimmune diseases, such as rheumatoid arthritis^73–75^, with common genetic influences explaining a substantial proportion of the observed co-occurrence^76^. NGF signaling was the final top pathway. NGF regulates a variety of neural processes such as differentiation, growth and survival of neurons, including hippocampal neurons, and is sensitive to stress. Specifically, NGF plays a role in the neuronal plasticity and survival of forebrain cholinergic neurons, which are memory-related^77,78^, thus NGF signaling might constitute a mechanism underlying memory consolidation abnormalities in PTSD^79^. Additional analyses, including the weighted gene co-expression network analysis^80^ (see Supplementary Materials), also identified several immune related ontologies, including neutrophil mediated immunity, neutrophil activation involved in immune response and neutrophil degranulation. Neutrophil has been shown to be a mediator of various diseases such as autoimmune diseases^81^ and psychological stress response^82^.

To investigate the practical utility of our gene expression findings, we constructed a PTSD polygenic expression score by aggregating 30 genes selected in the discovery sample using machine learning. The polygenic expression score achieved good accuracy to detect PTSD cases in the replication cohort (AUC = 0.764). It had modest specificity but high sensitivity, suggesting that this approach can become an informative screening tool. To evaluate the clinical utility of the polygenic expression score, we computed it in the past PTSD group and found expression level scores comparable to the current PTSD group, with both groups elevated relatively to the never PTSD group. This suggests that gene expression might be a potential biomarker that captures enduring vulnerability to, or a molecular scar following from PTSD, independent of current diagnostic status, and can provide clinically-relevant information not captured by the presenting concern of the patient. Future longitudinal studies are needed to establish whether the polygenic expression score can predict future PTSD. We also found a robust association between the score and dimensional measure of PTSD symptom severity in the replication sample. Although promising, the polygenic expression approach needs to be tested in other PTSD populations and for discriminating PTSD from depressive and anxiety disorders before its clinical utility is certain.

The finding that current and past PTSD groups show the same gene expression score levels can be interpreted in the broader context of the chronic course of PTSD in a significant proportion of patients^83–85^. PTSD recurrence may reflect an underlying biological vulnerability to this condition^86^. Our gene expression signature captures some of the pre-existing genetic vulnerability to PTSD that is independent of the genetic predisposition to the trauma exposure itself. Since exposure and PTSD show genetic correlation^87^ it is important that the present risk score captures unique risk for PTSD psychopathology, further informing the etiology of this condition. Thus, the score may constitute a novel marker of biological vulnerability to PTSD that may inform a screening tool useful for identifying at-risk individuals. Furthermore, gene expression captures environmental influences, and thus could additionally constitute a biological scar of experiencing PTSD. Perhaps such lasting biological changes following PTSD could be one of the mechanisms by which prior trauma may sensitize people to poorer response to later traumas, for example by leading to sustained alterations of the HPA axis^88,89^.

The current study had several strengths, including a state-of-the-art RNA-Seq approach, replication of results in an independent sample, and a common trauma in all participants, including exposed controls. Nonetheless, our findings must be considered in the context of several limitations. First, since our study is cross-sectional, we cannot determine whether observed alterations in the gene expression among PTSD participants are a consequence of the disorder or a part of its etiology. Comparisons to trauma-exposed controls suggest that differential gene expression is not just a consequence of trauma, but a longitudinal design is needed to determine the direction of the association of gene expression with PTSD. Second, our gene expression analysis was performed in RNA samples derived from whole blood and were thus a mix of cell types. We sought to control for the mix statistically, but future work needs to isolate and examine each cell type individually. Furthermore, not all genes and transcripts are expressed in the blood, and future studies should expand gene expression studies in PTSD to other tissues. Third, it is plausible that some discrepancies from previous studies are due to methodological differences, including sample characteristics and type of trauma exposure. It will be important to replicate our findings in larger, more diverse cohorts with other trauma experiences. Lastly, future pre-post studies are needed to evaluate whether the constructed polygenic expression score can predict onset and/or chronicity of PTSD after trauma exposure.

To conclude, the current study identified five genes differentially expressed in PTSD, including *FKBP5*. Together with the results of pathway analyses, these findings point to HPA-axis and immune dysregulation as key biological processes underpinning PTSD that may constitute potential biomarkers for this condition. We also derived a polygenic expression score that differentiates PTSD participants from trauma-exposed controls, that if validated in pre-post studies, would be a useful screening tool for research and clinical practice.

## Electronic supplementary material

Supplementary Methods

Supplementary Table 1

Supplementary Table 2

Supplementary Table 3

Supplementary Table 4

Supplementary Table 5

Supplementary Table 6

Supplementary Table 7

Supplementary Figure 1

Supplementary Figure 2

Supplementary Figure 3

Supplementary Figure 4
