# Supplementary material for: Gene expression associated with PTSD in World Trade Center responders: An RNA sequencing study
Source: Transl Psychiatry. 2017 Dec 18;7:1297. doi: 10.1038/s41398-017-0050-1 (PMC5802695; doi:10.1038/s41398-017-0050-1)
Supplement: Supplementary file 1 — Supplementary Methods [file 41398_2017_50_MOESM1_ESM.docx]

**Supplementary Methods**

*Paxgene total RNA isolation*

The purification of total RNA was prepared using the Paxgene Blood RNA kit (Qiagen).  2 ml human whole blood was collected in a PAXgene Blood RNA Tube, and frozen at -80C.  The tubes were then thawed at room temperature and incubated for 2 hours at room temperature to ensure complete lysis of blood cells.  Purification then began with a centrifugation step to pellet nucleic acids in the PAXgene Blood RNA Tube. The pellet was washed and resuspended. The resuspended pellet was incubated in optimized buffers together with proteinase K to bring about protein digestion. An additional centrifugation through the PAXgene Shredder spin column was carried out to homogenize the cell lysate and remove residual cell debris, and the supernatant of the flow through fraction was transferred to a fresh microcentrifuge tube. Ethanol was added to adjust binding conditions, and the lysate was applied to a PAXgene RNA spin column. During a brief centrifugation, RNA was selectively bound to the PAXgene silica membrane as contaminants pass through. Remaining contaminants were removed in several wash steps. Between the first and second wash steps, the membrane was treated with DNase I to remove trace amounts of bound DNA. After the wash steps, RNA was eluted in elution buffer (Buffer BR5) and heat-denatured.   Quantitative assessment of the purified total RNA was then accomplished by using a Qubit Broad Range RNA kit (Thermofisher).  The RNA was further evaluated qualitatively by a 2100 Bioanalyzer (Agilent technologies).

*RNA-Seq library preparation*

The sequencing libraries were prepared with the TruSeq Stranded Total RNA kit (Illumina Inc), from 1ug total RNA.  Following manufacturer’s instructions, the first step depleted rRNA from total RNA.  After ribosomal depletion, the remaining RNA was purified, fragmented and primed for cDNA synthesis.  Fragmented RNA was then reverse transcribed into first strand cDNA using random primers.  The next step removed the RNA template and synthesizes a replacement strand, incorporating dUTP in place of dTTP to generate ds cDNA. AMPure XP beads (Beckman Coulter) were used to separate the ds cDNA from the second strand reaction mix resulting in blunt-ended cDNA.  A single ‘A’ nucleotide was then added to the 3’ ends of the blunt fragments.  Multiple indexing adapters, containing a single ‘T’ nucleotide on the 3’ end of the adapter, were ligated to the ends of the ds cDNA, preparing them for hybridization onto a flow cell.  Adapter ligated libraries were amplified by PCR, purified using Ampure XP beads, and validated for appropriate size on a 4200 TapeStation D1000 Screentape (Agilent Technologies, Inc.).   The DNA libraries were quantitated using KAPA Biosystems qPCR kit, and were pooled together in an equimolar fashion, following experimental design criteria.  Each pool was denatured, diluted, and clustered to individual lanes of a HiSeq Flow Cell using an Illumina cBot and the corresponding single-read TruSeq cluster kit.  Pooled, clustered samples were then run on a HiSeq2500 sequencer according to the manufacturer's recommended protocol (Illumina Inc.).

*Agena iPLEX genotyping*

SNP genotyping was performed using iPLEX assay (Agena Bioscience, San Diego, CA). Briefly, the protocol involves PCR amplification of DNA using SNP specific primers, followed by a base extension reaction using the iPLEX Gold chemistry (Agena San Diego, CA). SNP- specific PCR and extension primers were designed and organized into pools with the Assay Design Suite (Agena). HotStar Taq Polymerase (Qiagen) was used for all PCRs. 15 ng of DNA was added to each 5-ul PCR reaction mixture a 384 well microtiter plate. The PCR condition was 94°C for 15 min for hot start, followed by 45 cycles of denaturing at 94°C for 20 sec, annealing at 56°C for 30 sec, extension at 72°C for 1 min for 45 cycles, and final incubation at 72°C for 3 min. The PCR products were then treated with SAP (shrimp alkaline phosphatase, Agena) for 40 min at 37°C then ramped to 85°C for 5 min to remove excess dNTPs, as described (23). iPLEX enzyme (Agena) was used for the base extension reactions. Base extension was carried out whereby both alleles interrogated by the base extension primer were extended by adding a mixture of ddNTPs and dNTPs. The final base extension products were diluted in 18 ul of double distilled water and then treated with 6mg of SpectroCLEAN (Agena) resin per well to remove contaminating salts. Treated extension product was spotted to the appropriate location on a 384-pad SpectroCHIP II (Agena) using a Sequenom MassARRAY Nanodispenser (Agena, San Diego, CA). A MassARRAY Analyzer Compact MALDI-TOF MS (Agena) was used for data acquisitions from the SpectroCHIP. The genotyping calls were generated in real time by the MassARRAY Typer Analyzer v4.0.26.73 (Agena). The call cluster plot for each SNP was viewed, and calls were manually adjusted if needed. Data was exported into a Microsoft Excel spreadsheet. Mendelian inconsistency was tested, and inconsistent assays were failed. The genotyping assay was performed by the Genomic Shared Resource of Roswell Park Cancer Institute.

*Cell type comparisons*

Two sample t-tests were applied to compare each cell type proportion versus PTSD status. Only CD4T showed p < 0.05 in the replication cohort (Supplementary Figure 2).

*Sensitivity analysis using gene exon counts*

Sensitivity analyses examined mature RNA alone (gene exon counts) in order to allow for comparison to papers in the literature that have only taken this approach. Differential expression analysis with gene exon counts identified 284 genes to be differentially expressed at FDR < 0.05 (Supplementary Table 1). Among the 284 genes, 96 exhibited p<0.05 in the replication cohort. Ten of these 96 genes have absolute fold change>1.2 in the discovery cohort. These genes were *CCDC85B, CMTM2, CRIPAK, GRB10, H1FX, H3F3C, HIST4H4, MARC1, NDUFA1* and *OMG*, of which *CRIPAK, GRB10, MARC1* and *OMG* were down-regulated in current PTSD. From the candidate gene analysis, 4 of the 20 genes (*FKBP5, SOD1, BBC3* and *C9orf84*) were significant at combined Bonferroni p<0.05 (Supplementary Table 2). Pathway analysis among the 284 differentially expressed genes in the discovery cohort identified the glucocorticoid receptor signaling pathway at FDR < 0.05 after adjusting for multiple comparisons (Supplementary Table 3). The polygenic expression score was trained on 284 genes at FDR<0.05 from the discovery cohort. The final polygenic expression score from the elastic net algorithm retained 26 genes (Supplementary Table 4) and achieved AUC=0.726 in the replication cohort. Polygenic expression score was significantly correlated with PCL in the replication sample (r=0.282, p<0.01). The past group exhibited the same level of expression score as the current group (p=0.219), and the scores of each group were significantly different than the never group (p<0.01 for both), consistent with the findings from gene body counts analysis.

*Differential expression analysis in past PTSD*

A 3-group (current, past and never) analysis was carried out to evaluate the group effect via a likelihood ratio test. Among the genes which exhibited significant group effect, post-hoc pairwise comparison showed that these genes were driven by the difference between current PTSD and never. Only one gene (*GRB10*) was driven by both current PTSD vs never and past PTSD vs never. No genes was significantly associated with current vs past PTSD. We also reran the pairwise differential expression analysis using the Wald test. Differential expression analysis of current PTSD in the discovery cohort versus past PTSD did not identify any significant genes at FDR < 0.05. On the other hand, comparison of past PTSD and never PTSD identified *GRB10* to be up-regulated at FDR < 0.05 using gene body counts, whereas no gene was identified using gene exon counts. This could be attributed to insufficient power of our past PTSD sample size (n = 42) and the heterogeneity among the past PTSD subjects in terms of timing of onsets and remissions as well as current symptom severity.

*PTSD versus other clinical comorbidities*

PTSD is often comorbid with a range of mental disorders, as well as physical health problems^1^. PTSD is most prominently comorbid with depression (ref), and comorbid MDD was assessed in our sample using a diagnostic interview. During the monitoring visits, participants’ lower respiratory disease (LRD), upper respiratory disease (URD), anxiety and gastroesophaheal reflux disease (GERD) were also assessed. These are hallmark WTC illnesses and often co-occur with PTSD ^1^. We used these comorbid disorders and checked whether (a) they were higher in cases than in controls, and (b) the gene expression score obtained for PTSD was still associated PTSD after adjusting for each comorbid disorder, via linear regression models using the gene expression score as outcome in the replication cohort. As expected given the high comorbidity, all other disorders that we looked at were significantly elevated in PTSD cases than in control, except for URD in the replication cohort (Supplementary Table 7). On the other hand, the gene expression score was still significantly associated with PTSD (p < 0.05) after adjusting for each comorbidity.

*Weighted gene co-expression network analysis*

The weighted gene co-expression network analysis (WGCNA) ^2^ was used to identify modules of correlated genes. The WGCNA algorithm was performed on log transformed normalized gene body read counts. The Pearson correlation matrix was raised to the sixth power to achieve scale free topology in the discovery cohort. The minimum module size was set as 30, and the cut-offs for splitting and merging modules were 2 and 0.25, respectively. 22 modules were identified from the discovery cohort. The gene expression profiles for each module were represented by the eigengene. The association between module eigengene and PTSD was performed using linear regression, adjusting for age, race and cell type proportions, and significant modules were identified using FDR 0.1. 5 modules were identified at FDR < 0.1. All the five genes (*FKBP5, NDUFA1, CCDC85B, SNORD54, SNORD46*) that was identified from differential expression analysis were in the largest module (turquoise) which contained 3860 genes. The other 4 significant modules were of sizes 417 (black), 80 (grey60), 377 (pink) and 439 (red), respectively. Bioconductor package clusterProfiler ^3^ was used to identify enriched KEGG pathways and gene ontologies among the genes in these 4 modules. The minimum and maximum gene set size were set as 15 and 500, respectively. The top 10 gene sets within each ontology for each module were reported in Supplementary Table 6. The pink module identified several immune related ontologies, namely neutrophil mediated immunity, neutrophil activation involved in immune response and neutrophil degranulation.

**References**

1. Luft B, Schechter C, Kotov R, Broihier J, Reissman D, Guerrera K *et al.* Exposure, probable PTSD and lower respiratory illness among World Trade Center rescue, recovery and clean-up workers. *Psychological medicine* 2012; **42**(05)**:** 1069-1079.

2. Langfelder P, Horvath S. WGCNA: an R package for weighted correlation network analysis. *BMC Bioinformatics* 2008; **9:** 559.

3. Yu G, Wang L-G, Han Y, He Q-Y. clusterProfiler: an R package for comparing biological themes among gene clusters. *Omics: a journal of integrative biology* 2012; **16**(5)**:** 284-287.

4. Logue MW, Smith AK, Baldwin C, Wolf EJ, Guffanti G, Ratanatharathorn A *et al.* An analysis of gene expression in PTSD implicates genes involved in the glucocorticoid receptor pathway and neural responses to stress. *Psychoneuroendocrinology* 2015; **57:** 1-13.

**Supplementary Figure Legend**

**Supplementary Figure 1:** Overview of the RNA-Seq data analysis pipeline.

**Supplementary Figure 2:** Estimated cell type proportions in A. discovery cohort and B. replication cohort, respectively. Two-sample t-tests p-values are printed above the bar for each cell type.

**Supplementary Figure 3:** Scatter plot comparing the predicted polygenic expression score in replication cohort against PCL score. The estimated Pearson correlation coefficient is 0.315 (p = 0.00315).

**Supplementary Figure 4:** Bar plots comparing the FKBP5 gene expression, stratified by genotypes in SNPs A. rs9296158, B. rs1360780, C. rs3800373, D. rs9470080 in discovery cohort and SNPs E. rs9296158, F. rs1360780, G. rs3800373, H. rs9470080 in replication cohort.

**Supplementary Table Legends**

**Supplementary Table 1:** List of 448 and 284 genes at FDR < 0.05 identified from the discovery cohort from the gene body and gene exon counts analysis, respectively. The log2 fold change, nominal p-value and FDR for each gene in both the discovery and replication cohort are provided. Genes with nominal p-value < 0.05 in the replication cohort, large absolute fold change (> 1.2) and consistent fold change estimates are marked with asterisks in the last two columns.

**Supplementary Table 2:** List of 35 candidate genes compiled by Logue et al. (2015)^4^.

**Supplementary Table 3:** List of IPA canonical pathways tested among the differentially regulated genes from the gene body and gene exon counts analysis, respectively. The Fisher’s p-value and the overlapping genes are provided.

**Supplementary Table 4:** List of genes and the estimated coefficients retained in the prediction model from the gene body and gene exon counts analysis, respectively.

**Supplementary Table 5:** Association between genotypes and PTSD in discovery and replication cohorts. The p-values are computed from chi-squared tests.

**Supplementary Table 6:** Top 10 KEGG pathways, biological process (BP), cellular component (CC) and molecular function (MF) for each module and the corresponding FDR values.

**Supplementary Table 7:** Clinical comorbidities of samples in discovery and replication cohorts. The p-values were computed from one way analysis of variance (for continuous clinical comorbidities in all samples), t-test (for continuous clinical comorbidities in discovery/replication cohort comparing current to never) and chi-squared test (for categorical clinical comorbidities).
