# Supplementary figures and images for: Gene expression associated with PTSD in World Trade Center responders: An RNA sequencing study
Source: Transl Psychiatry. 2017 Dec 18;7:1297. doi: 10.1038/s41398-017-0050-1 (PMC5802695; doi:10.1038/s41398-017-0050-1)

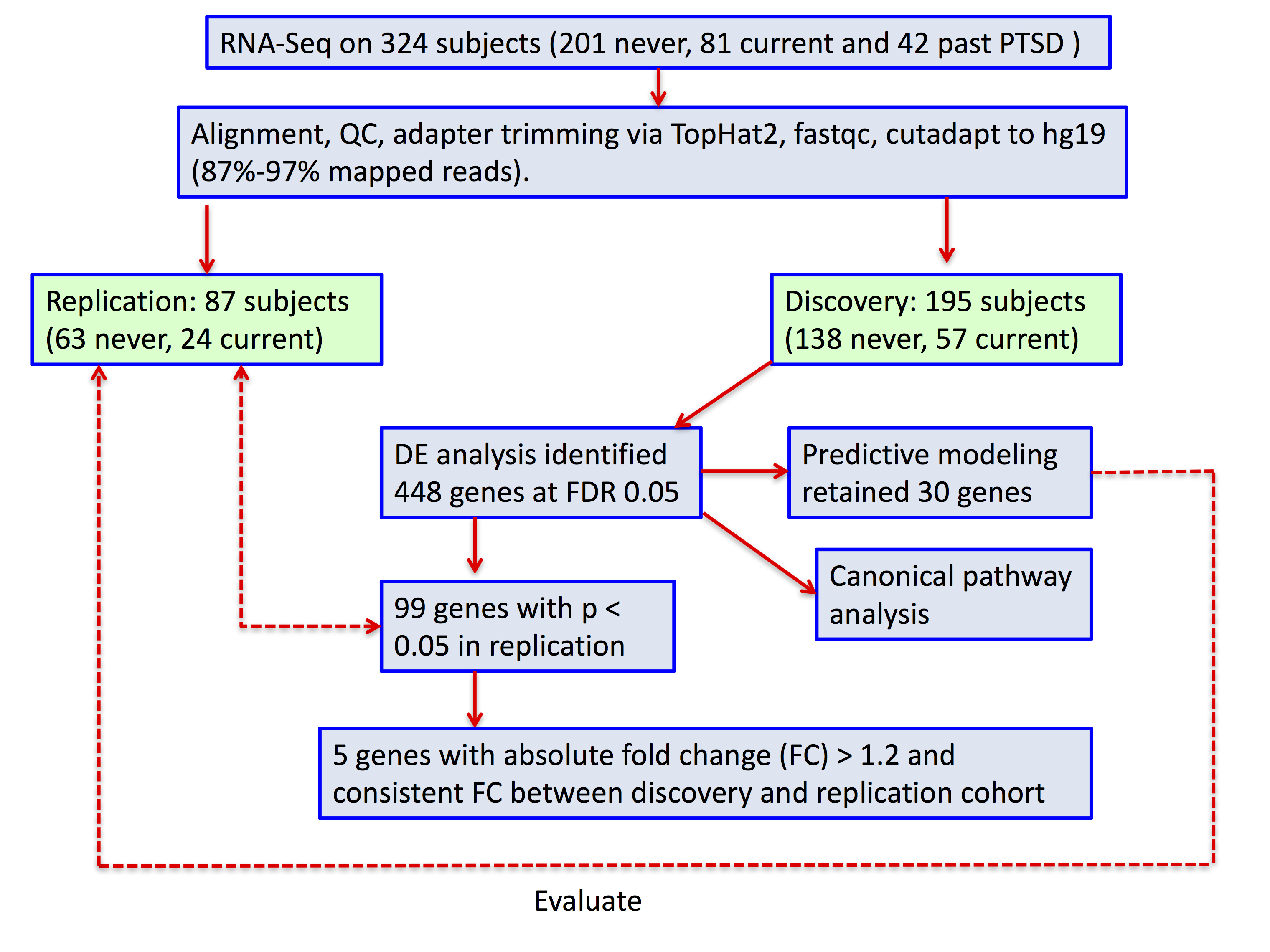

Supplement: Supplementary file 9 — Supplementary Figure 1 [file 41398_2017_50_MOESM9_ESM.jpg]

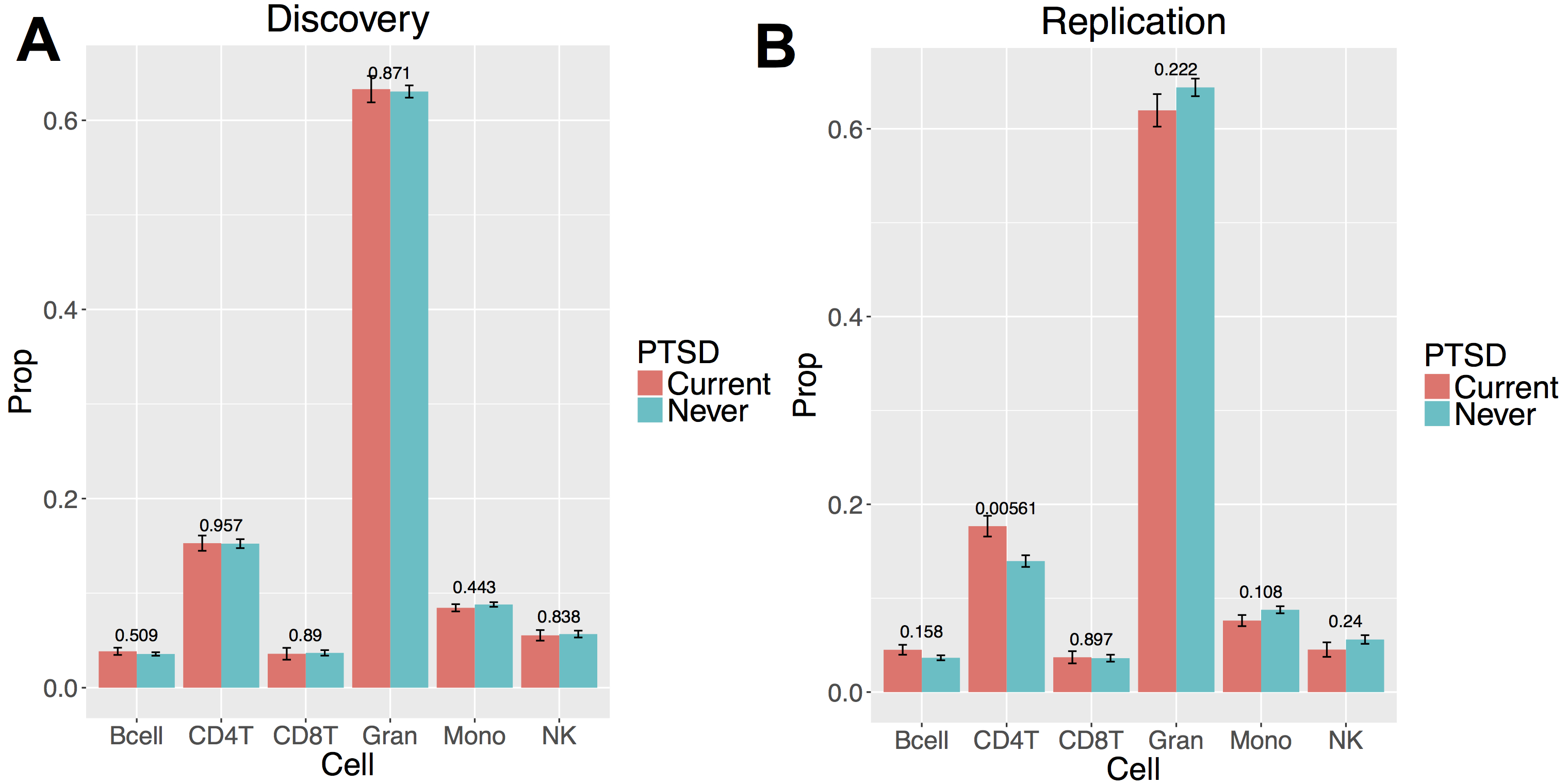

Supplement: Supplementary file 10 — Supplementary Figure 2 [file 41398_2017_50_MOESM10_ESM.jpg]

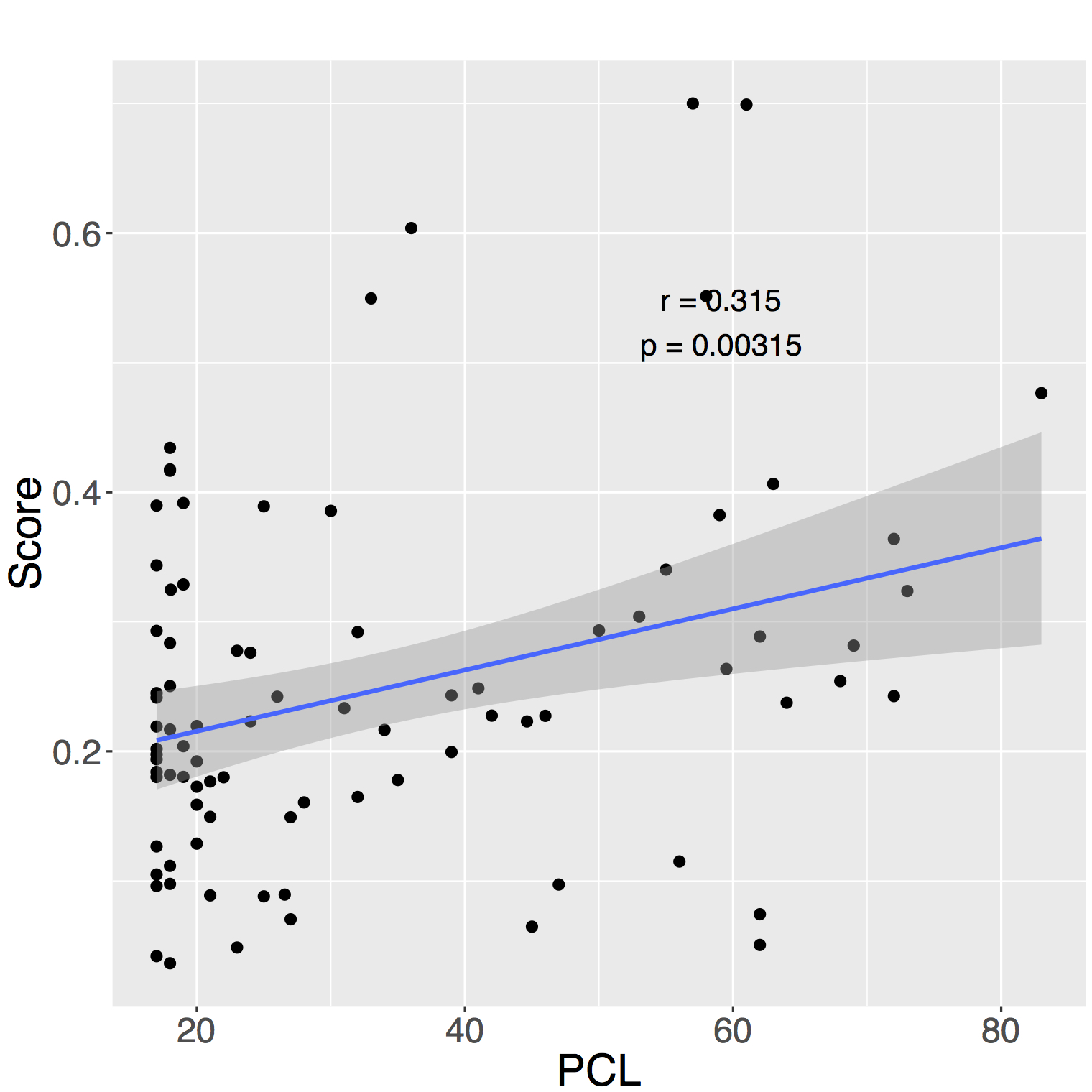

Supplement: Supplementary file 11 — Supplementary Figure 3 [file 41398_2017_50_MOESM11_ESM.jpg]

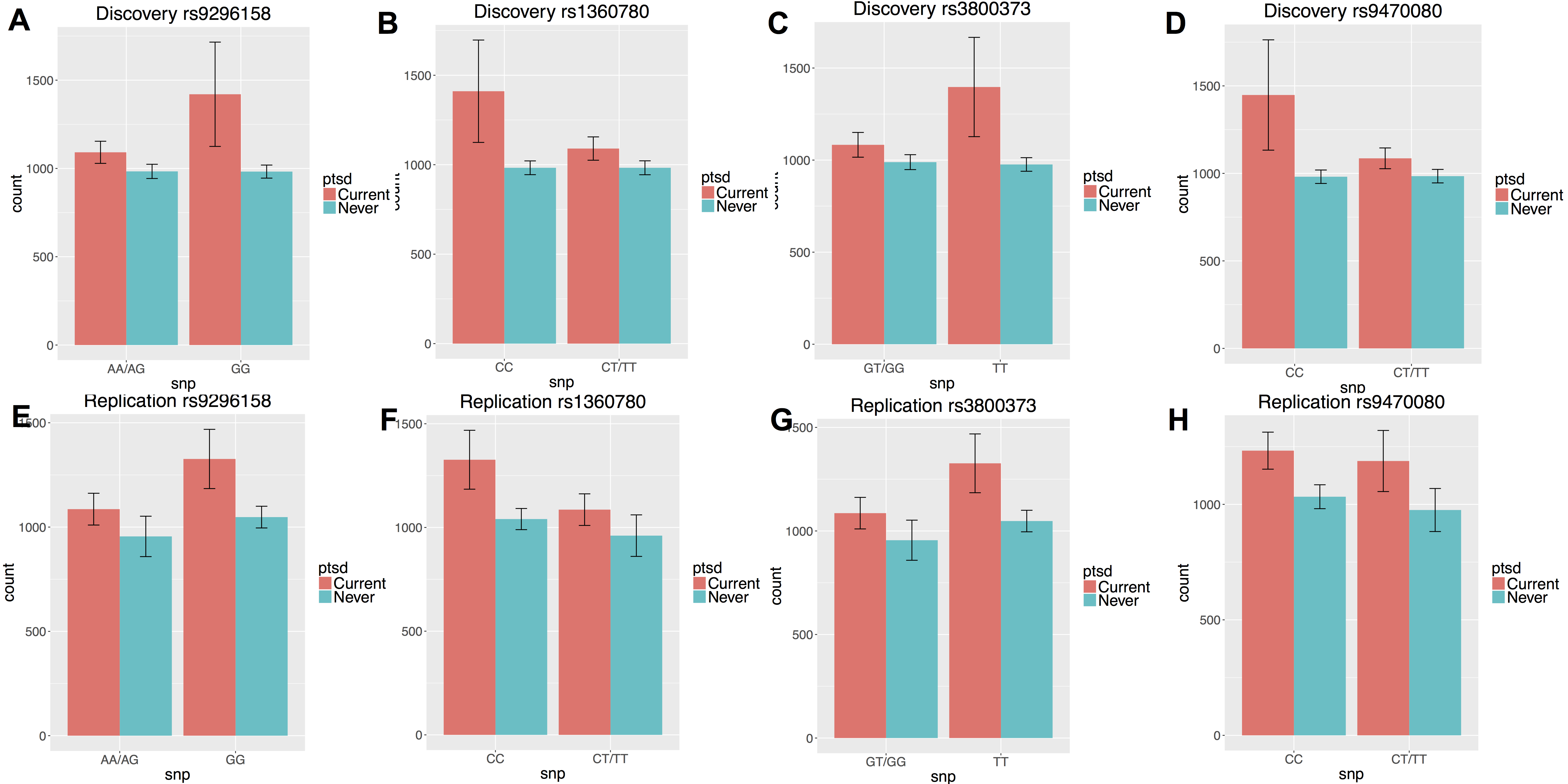

Supplement: Supplementary file 12 — Supplementary Figure 4 [file 41398_2017_50_MOESM12_ESM.jpg]
